# Room‐Temperature Magnetic Skyrmions and Intrinsic Anomalous Hall Effect in a Nodal‐Line Kagomé Ferromagnet MnRhP

**DOI:** 10.1002/advs.202521734

**Published:** 2026-01-21

**Authors:** Kosuke Karube, Ming‐Chun Jiang, Lukas Keller, Jonathan S. White, Yi Ling Chiew, Xiuzhen Yu, Guang Yu Guo, Ryotaro Arita, Yoshinori Tokura, Yasujiro Taguchi

**Affiliations:** ^1^ RIKEN Center for Emergent Matter Science (CEMS) Wako Japan; ^2^ Department of Physics and Center for Theoretical Physics National Taiwan University Taipei Taiwan; ^3^ Paul Scherrer Institute (PSI) Villigen Switzerland; ^4^ Physics Division National Center for Theoretical Sciences Taipei Taiwan; ^5^ Department of Physics University of Tokyo Bunkyo‐ku Tokyo Japan; ^6^ Department of Applied Physics University of Tokyo Bunkyo‐ku Japan; ^7^ Tokyo College University of Tokyo Bunkyo‐ku Japan

**Keywords:** anomalous Hall effect, berry curvature, kagomé lattice, skyrmions

## Abstract

Topological magnetic semimetals with kagomé lattices have attracted significant attention due to their nontrivial electronic band structures and pronounced electromagnetic responses. The search for kagomé‐lattice topological semimetals exhibiting magnetic ordering above room temperature is essential for advancing their potential in device applications. In this work, we report direct observations of topological magnetic textures and anomalous Hall effects driven by topological nodal lines in MnRhP, a room‐temperature ferromagnet with a distorted kagomé lattice. Using single‐crystal magnetization measurements and powder neutron diffraction, we reveal a weak uniaxial magnetic anisotropy. Lorentz transmission electron microscopy observations confirm the presence of stable magnetic skyrmions above room temperature. Moreover, both the ordinary and anomalous Hall effects are significantly enhanced upon cooling, with a large anomalous Hall conductivity (AHC) observed at low temperatures. First‐principles calculations indicate significant contributions to electronic states near the Fermi level from both in‐plane and out‐of‐plane *d* orbitals of Mn and Rh, resulting in the low magnetic anisotropy energy. The calculated Berry curvature reproduces the experimentally observed large AHC, providing direct evidence for an intrinsic mechanism linked to the topological nodal lines. These findings establish MnRhP as a promising kagomé‐lattice magnet for investigating topological magnetic textures and anomalous transport phenomena at room temperature.

## Graphical abstract

MnRhP has been identified as a novel kagomé magnet that hosts magnetic skyrmions above room temperature and exhibits a large intrinsic anomalous Hall effect. The latter originates from Berry curvature associated with gapped nodal lines, establishing MnRhP as a promising platform for exploring high‐temperature topological phenomena in both real and momentum spaces.

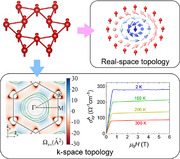

## 1 Introduction

Topological semimetals have attracted much attention as a new class of quantum materials characterized by nontrivial band crossings around the Fermi level [1, 2]. They are broadly categorized into Dirac and Weyl types, where the band crossings occur at points in momentum space, and nodal‐line types, where the band crossings form continuous lines or rings. In magnetic systems, these topological band crossings generate large Berry curvature, which leads to unconventional and enhanced transport phenomena, such as large intrinsic anomalous Hall effects [3]. Thus far, numerous magnetic topological semimetals have been reported [4, 5, 6, 7, 8, 9, 10, 11, 12, 13, 14, 15, 16, 17, 18, 19, 20, 21, 22, 23, 24, 25]. Among them, magnetic materials with kagomé‐lattice structures, such as Mn_3_Sn [4, 5], FeSn [6, 7], Fe_3_Sn_2_ [8, 9], Fe_3_Sn [10, 11], Co_3_Sn_2_S_2_ [12, 13, 14, 15] and *R*Mn_6_Sn_6_ (*R*: rare earth) [16, 17], have attracted significant interest due to their geometric frustration, topological and flat bands, and relatively high magnetic ordering temperatures. Nevertheless, the search for new kagomé‐lattice topological semimetals exhibiting magnetic ordering above room temperature remains a major challenge in condensed matter physics. These materials are indispensable not only for deepening the fundamental understanding of topological electronic states but also for enabling future applications in spintronics and quantum devices. In particular, it is highly desirable to identify kagomé materials that can host topological magnetic structures, such as magnetic skyrmions, above room temperature, while simultaneously exhibiting a large intrinsic anomalous Hall response for diverse device applications. However, such materials are exceedingly scarce, with only a few examples, including Fe_3_Sn_2_ [26] and TbMn_6_Sn_6_ [27].

**FIGURE 1:**
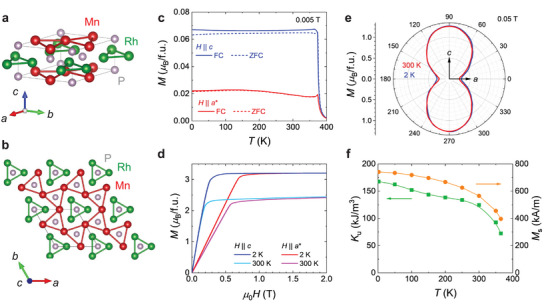
Crystal structures and magnetometry measurements. (a,b) Crystal structure of MnRhP, constructed from structural data obtained from single‐crystal X‐ray diffraction measurements (see Table S1,). (c) Temperature dependence of magnetization measured under a magnetic field of 0.005 T applied along the a^*^‐axis and *c*‐axis. Field‐cooling (FC) and zero‐field‐cooling (ZFC) data are shown by solid and dashed lines, respectively. (d) Magnetic field dependence of magnetization along the a^*^‐axis and *c*‐axis at 300 and 2 K. (e) Field‐orientation dependence of magnetization under a 0.05 T magnetic field rotated within the *ac*‐plane, measured at 300 and 2 K. (f) Temperature dependence of the uniaxial magnetic anisotropy constant *K*_u_ (green) and saturation magnetization *M*_s_ (orange), where 100 kA/m corresponds to 0.432 *µ*_B_/f.u. The density (7.81 g/cm^3^), obtained from single‐crystal X‐ray diffraction, was used to convert from mass units to volume units.

In this work, we report the discovery of both room‐temperature skyrmions and a large anomalous Hall effect in the novel kagomé ferromagnet MnRhP, a promising candidate for a topological nodal‐line semimetal. MnRhP belongs to the broad family of ternary transition metal pnictides with the formula TT'X (T, T’ = transition metals, X = P, As). [28] Among these compounds, MnRhP crystallizes in the ZrNiAl‐type (also known as the ordered Fe_2_P‐type) noncentrosymmetric hexagonal structure, with space group *P* $\bar{6}$2*m*, where Mn atoms form a distorted kagomé lattice, as illustrated in Figure 1a,b. This unique lattice geometry is known to host diverse physical phenomena, such as superconductivity in nodal‐line semimetals (Zr, Hf)RuP [29] and CaAgP [30, 31], frustrated spin‐ice states in HoAgGe [32], and quantum critical behaviors in CeRhSn [33] and UCoAl [34].

MnRhP itself has long been known as a room‐temperature ferromagnet with a high Curie temperature of *T*_c_ ∼ 400 K [28, 35, 36, 37]. Earlier neutron diffraction experiments reported that the easy magnetization axis is tilted by 26 degrees from the *c*‐axis [37]. More recently, Singh et al. used first‐principles calculations to predict the presence of a topological nodal line near the Fermi level in MnRhP [38]. Their calculations further suggested that the magnetization aligns along the *a*‐axis, which breaks horizontal mirror symmetry and results in the transformation of the nodal line into Weyl nodes. These studies suggest that MnRhP is a novel kagomé‐lattice topological semimetal exhibiting robust ferromagnetism above room temperature. However, the precise magnetic structure remains unclear, particularly the direction of the easy axis, given the discrepancy between earlier neutron data [37] and recent theoretical predictions [38]. Moreover, although a large intrinsic anomalous Hall conductivity (AHC) was theoretically predicted as a result of the Berry curvature [38], no experimental verification has been reported. Therefore, further experimental investigations using single crystals, combined with theoretical calculations, are necessary to resolve these inconsistencies and to fully understand the magnetic and topological properties of MnRhP.

In this work, we successfully synthesized high‐quality single crystals of MnRhP using a self‐flux method. Single‐crystal magnetometry and powder neutron diffraction measurements revealed that the easy magnetization axis lies along the *c*‐axis, although the uniaxial magnetic anisotropy energy is relatively weak. Using Lorentz transmission electron microscopy (LTEM), we directly observed stable magnetic skyrmions persisting above room temperature, which is attributed to the combination of dipolar interaction and uniaxial anisotropy. In addition, temperature‐dependent transport measurements on single crystals revealed both large ordinary and anomalous Hall effects. Our first‐principles calculations account for the observed AHC, supporting an intrinsic mechanism driven by Berry curvature associated with topological nodal lines.

## 2 Results and Discussions

### 2.1 Singe‐Crystal Magnetometry

Bulk single crystals of MnRhP (Figure S1) were synthesized using a self‐flux method, as described in Methods. The ZrNiAl‐type hexagonal crystal structure, characterized by distorted Mn kagomé layers (Figure 1b), was confirmed by single‐crystal X‐ray diffraction (Table S1). Magnetization measurements were performed on a single crystal with equal demagnetization factors along the *a* ^*^‐axis and *c*‐axis. As shown in Figure 1c, the sample exhibits a ferromagnetic transition at *T*_c_ ∼ 375 K. Field‐dependent magnetization curves at 300 and 2 K along both axes are shown in Figure 1d. These curves reveal that the *c*‐axis is magnetically easier. To further identify the magnetic anisotropy direction, we performed field (0.05 T)‐orientation‐dependent magnetization measurements by rotating the single crystal within the *ac*‐plane. As shown in Figure 1e, the magnetization is maximized along the *c*‐axis and minimized along the *a*‐axis, confirming that the *c*‐axis is the easy axis while the *a*‐axis is the hard axis. This result rules out the presence of a canted magnetization.

Figure 1f displays the temperature dependence of saturation magnetization (*M*_s_) and the uniaxial magnetic anisotropy constant (*K*_u_). *K*_u_ was estimated from the integrated area between the magnetization curves along the *c*‐axis and *a*‐axis shown in Figure 1d. The saturation magnetization at 2 K is *M*_s_ ∼ 3.2 *µ*_B_/f.u. (∼ 740 kA/m), which is consistent with previous reports [36]. The anisotropy constant is *K*_u_ ∼ 168 kJ/m^3^ at 2 K and *K*_u_ ∼ 125 kJ/m^3^ at 300 K. These values are significantly smaller than those of typical hard uniaxial magnets, such as Fe_2_P (*K*_u_ ∼ 2.3 MJ/m^3^ at 4 K) [39, 40] with the same hexagonal crystal structure. The quality factor, defined as $Q=2K_{u}/\left(\mu_{0}M_{s}^{2}\right)$, is estimated to be 0.5 at 2 K and 0.6 at 300 K. These results demonstrate that MnRhP is a soft ferromagnet with large demagnetization and relatively weak uniaxial anisotropy.

### 2.2 Powder Neutron Diffraction

The uniaxial magnetic anisotropy along the *c*‐axis identified in our magnetometry measurements differs from a previous neutron diffraction study, which reported that the easy axis is tilted by 26 degrees from the *c*‐axis [37]. To independently verify our findings, we conducted zero‐field powder neutron diffraction measurements over a broad temperature range (see Methods for details).

**FIGURE 2:**
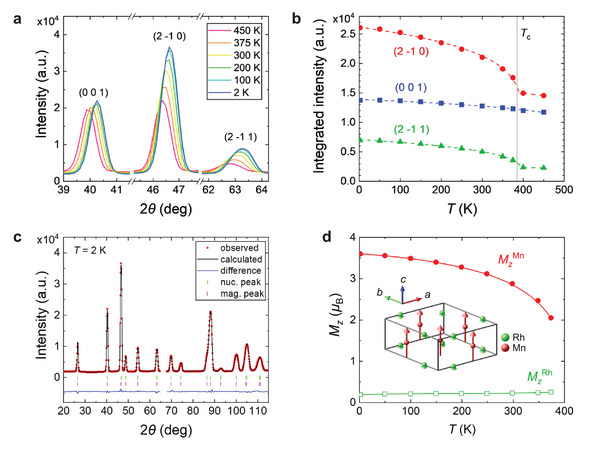
Powder neutron diffraction measurements. (a) Neutron diffraction intensity profiles focusing on the (0 0 1), (2 −1 0) and (2 −1 1) reflections at selected temperatures. (b) Temperature dependence of the integrated intensity of the (0 0 1), (2 −1 0) and (2 −1 1) peaks. (c) Neutron diffraction profile at 2 K and the refinement of both nuclear and magnetic reflections. Data points around 2*θ* ∼ 66° were excluded from the refinement due to extrinsic scattering originating from the sample environment. (d) Temperature dependence of the magnetic moments along the *c*‐axis for Mn and Rh atoms obtained from the refinement. The inset illustrates the schematic ferromagnetic structure determined from the refinement at 2 K.

Figure 2a shows neutron diffraction patterns around the nuclear reflections (0, 0, 1), (2, −1, 0), and (2, −1, 1) measured at various temperatures. The intensities of the (2, −1, 0) and (2, −1, 1) reflections increase sharply with decreasing temperature, while the (0, 0, 1) reflection shows only a weak temperature dependence. As plotted in Figure 2b, the integrated intensities of the (2, −1, 0) and (2, −1, 1) peaks exhibit a sudden increase below the ferromagnetic transition temperature *T*_c_ ∼ 375 K, in contrast to the nearly constant intensity of the (0, 0, 1) peak. As the intensity of the magnetic peak represents the magnitude of magnetic moments perpendicular to the magnetic scattering vector, this behavior indicates the emergence of ferromagnetic ordering with magnetic moments aligned along the *c*‐axis.

To further quantify the magnetic structure, we performed systematic magnetic structural refinements. The magnetic structure can be expressed as a linear combination of magnetic irreducible representations, Γ = 1Γ_2_ + 1Γ_3_ + 1Γ_4_ + 1Γ_5_ + 2Γ_6_, where Γ_2_ corresponds to ferromagnetism with moments along the *c*‐axis, and Γ_3_ to Γ_6_ represent various magnetic arrangements in the *ab*‐plane. This formulation accounts for all symmetry‐allowed magnetic configurations within the space group *P* $\bar{6}$2*m* and the magnetic propagation vector *Q* = (0, 0, 0). The final refinement of the diffraction pattern at 2 K is shown in Figure 2c. The results demonstrate that the magnetic structure is fully described by the Γ_2_ component, and the *ab*‐plane components (Γ_3_−Γ_6_) are zero within experimental error, confirming that the magnetic moments are strictly aligned along the *c*‐axis. The refinement also reveals that the ferromagnetism is predominantly carried by the Mn moments, while the Rh moments are only weakly polarized, aligning parallel to the Mn moments (Figure 2d). The temperature dependence of the Mn moment, as shown in Figure 2d, is consistent with the saturation magnetization in Figure 1f. These findings support the magnetometry results shown in Figure 1e and clearly demonstrate that MnRhP exhibits uniaxial magnetic anisotropy along the *c*‐axis without any tilting, although the anisotropy energy is small.

While MnRhP exhibits simple *c*‐axis collinear ferromagnetism, other MnT'X compounds display various complex magnetic structures that strongly depend on the Mn–Mn distances and the electronic structure determined by the combination of T’ and X elements, as discussed in (Table S2 and Figure S2).

### 2.3 LTEM Observations of Magnetic Skyrmions

**FIGURE 3:**
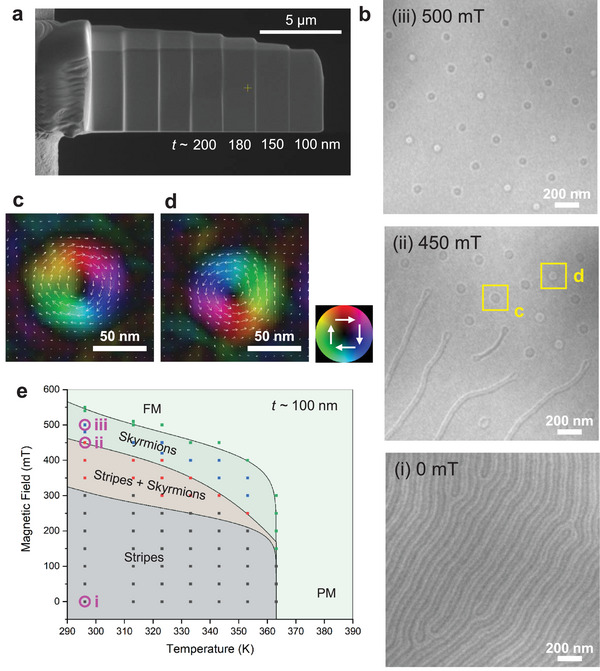
LTEM measurements on a thin plate. (a) Scanning electron microscopy (SEM) image of the thin plate with various thickness (*t*). (b) Under‐focused LTEM images observed in the region with *t* ∼ 100 nm at 296 K under magnetic fields of (i) 0 mT, (ii) 450 mT and (iii) 500 mT. (c,d) In‐plane magnetic induction field maps of skyrmions in the yellow boxes in panel b‐(ii), obtained from transport‐of‐intensity equation (TIE) analysis. A hue‐saturation‐lightness color wheel with white arrows is shown to indicate the in‐plane magnetic induction directions. (e) Temperature‐magnetic field phase diagram of the magnetic structures revealed by LTEM observations. The corresponding positions of the LTEM images in panels b (i, ii, and iii) are indicated in the phase diagram.

The competition between demagnetization energy and domain‐wall energy can stabilize a variety of magnetic domain structures, including magnetic skyrmions carrying topological charge. To investigate the formation of skyrmions in MnRhP, we performed LTEM on a thin‐plate sample, as shown in Figure 3a, whose normal is parallel to the *c*‐axis. The LTEM images observed in a region with a thickness of 100 nm at 296 K under different magnetic fields are shown in Figure 3b. As shown in Figure 3b(i), magnetic stripe domains are observed at room temperature under zero magnetic field. Upon applying a magnetic field perpendicular to the plate, the stripe domains gradually become fragmented and evolve into isolated magnetic bubbles around 450 mT, as shown in Figure 3b(ii). Magnetic‐induction mapping obtained by transport‐of‐intensity equation (TIE) analysis (Figure 3c,d) reveals that these magnetic bubbles correspond to Bloch‐type skyrmions. Notably, skyrmions with opposite helicities, as evidenced by the dark and bright contrast in circular disks, coexist. The opposite helicities suggest that dipolar interactions play a dominant role in stabilizing the skyrmions, rather than the Dzyaloshinskii–Moriya interaction. The domain wall thickness, indicated by the length of the radial direction of the colored area in the TIE image (Figure 3c,d), is approximately estimated to be 40 nm, which is relatively close to half of the skyrmion diameter (∼ 95 nm). This ratio reflects the weak uniaxial anisotropy energy in MnRhP, consistent with a quality factor *Q* < 1. With further increase in magnetic field, the stripe domains completely transform into skyrmions at around 500 mT, as shown in Figure 3b(iii). The magnetic phase diagram, constructed from LTEM imaging at various temperatures and magnetic fields, is shown in Figure 3e. A stable skyrmion phase exists over a broad temperature‐field region above room temperature. These results demonstrate that MnRhP is a promising system for realizing robust magnetic skyrmions above room temperature.

### 2.4 Transport Measurements

**FIGURE 4:**
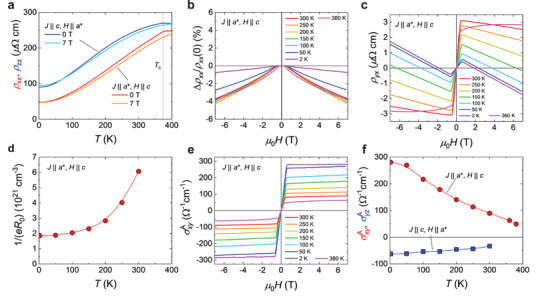
Electrical transport measurements. (a) Temperature dependence of the longitudinal resistivity *ρ_xx_* (*J* || *a* ^*^, *H* || *c*) and *ρ_zz_* (*J* || *c*, *H* || *a* ^*^) measured at 0 T and 7 T. b, c)　Magnetic field dependence of (b) the magnitude of magnetoresistance Δ*ρ_xx_*/*ρ_xx_* (0) and (c) the Hall resistivity *ρ_yx_* (*J* || *a* ^*^, *H* || *c*). (d) Temperature dependence of the nominal carrier density, estimated as 1/(*eR*_0_), where *R*_0_ is the ordinary Hall coefficient. (e) Magnetic field dependence of the anomalous Hall conductivity *σ_xy_* ^A^ (*J* || *a* ^*^, *H* || *c*). (f) Temperature dependence of anomalous Hall conductivity *σ_xy_* ^A^ (*J* || *a* ^*^, *H* || *c*) and *σ_yz_* ^A^ (*J* || *c*, *H* || *a* ^*^) measured at 7 T.

Electrical transport measurements were carried out on bulk single crystals of MnRhP in two configurations: (*J* || *a* ^*^, *H* || *c*) and (*J* || *c*, *H* || *a* ^*^). Figure 4a shows the temperature dependence of the longitudinal resistivity at zero field and an applied field of 7 T for both *ρ_xx_* (*J* || *a* ^*^, *H* || *c*) and *ρ_zz_* (*J* || *c*, *H* || *a* ^*^). In both orientations, the resistivity exhibits a broad maximum near the ferromagnetic transition temperature *T*_c_ ∼ 375 K and gradually decreases upon cooling. This behavior is consistent with previous reports on polycrystalline samples [36]. As shown in Figure 4b, negative magnetoresistance (MR) is observed in *ρ_xx_* over the entire temperature range, where the largest MR appears near *T*_c_.

Figure 4c presents the field dependence of the Hall resistivity *ρ_yx_* measured in the (*J* || *a* ^*^, *H* || *c*) configuration. The total Hall resistivity is expressed as the sum of the ordinary Hall resistivity (*ρ_yx_* ^0^ = *R*_0_ *µ*_0_ *H*), where *R*_0_ is the ordinary Hall coefficient, and the anomalous Hall resistivity (*ρ_yx_* ^A^). *R*_0_ was determined by linear fitting of the Hall resistivity in the high‐field region, and the nominal carrier density, estimated as (*eR*_0_)^−1^, is plotted as a function of temperature in Figure 4d. The nominal carrier density decreases substantially upon cooling, reaching 2 × 10^21^ cm^−3^ at 2 K.

The anomalous Hall resistivity was extracted by subtracting the linear ordinary Hall component from the total Hall resistivity. The corresponding anomalous Hall conductivity (AHC), defined as *σ_xy_* ^A^ ∼ *ρ_yx_* ^A^/*ρ_xx_* ^2^, is shown as a function of magnetic field in Figure 4e. The temperature dependence of *σ_xy_* ^A^ at 7 T is shown in Figure 4f. The AHC increases significantly upon cooling, reaching *σ_xy_* ^A^ ∼ 280 Ω^−1^cm^−1^ at 2 K. The corresponding anomalous Hall angle (AHA) is *σ_xy_* ^A^/*σ_xx_* ∼ 0.013. These large magnitudes of AHC and AHA are typical for intrinsic anomalous Hall effects in ferromagnetic topological semimetals [8, 9, 11, 16, 21, 22]. Indeed, as detailed in the following section, the observed AHC agrees with the theoretical value obtained from Berry‐curvature calculations, confirming its intrinsic origin. Furthermore, the corresponding longitudinal conductivity at 2 K, *σ_xx_* ∼ 2.1 × 10^4^ Ω^−1^cm^−1^, falls within the intrinsic regime rather than the highly conductive extrinsic regime [41].

The pronounced enhancement of the AHC upon cooling is much steeper than the gradual increases in the saturation magnetization. This strong temperature dependence is likely due to substantial modifications of the band structure with non‐trivial band crossings near the Fermi level arising from the large spin splitting of the Mn *d* orbitals, as discussed in the theory section. This interpretation is also consistent with the significant reduction in the nominal carrier density upon cooling, as shown in Figure 4d.

For comparison, the AHC measured in an alternative geometry (*J* || *c*, *H* || *a* ^*^), where *σ_yz_* ^A^ ∼ *ρ_zy_* ^A^/(*ρ_yy_ρ_zz_*), is also plotted in Figure 4f (see Figure S2 for the field dependence of *ρ_zz_* and *ρ_zy_*). In this configuration, the AHC is negative, with *σ_yz_* ^A^ ∼ −63 Ω^−1^cm^−1^ at 2 K. The opposite sign of the AHC between the two geometries is also confirmed by our theoretical calculations (Figure S6), which can be attributed to the anisotropic Berry curvature arising from the electronic band structure in the presence of spin‐orbit coupling (SOC). The roles of Berry curvature and SOC will be quantitatively discussed in the following theoretical section.

We finally note the absence of apparent topological Hall signals in our transport data. Although nanoscale skyrmions were observed in a thin‐plate specimen by LTEM, the skyrmion size in the bulk crystal used for the Hall measurements expands to several micrometers due to the dominant dipolar interaction, which significantly suppresses the emergent magnetic field. As a result, the expected topological Hall resistivity becomes orders of magnitude smaller than the anomalous Hall resistivity.

### 2.5 First‐Principles Calculations

To elucidate the microscopic origin of magnetism and the large AHC in MnRhP, we performed first‐principles calculations based on the density functional theory (DFT) for the ferromagnetic state, assuming that the Mn moments are aligned along the *c*‐axis. The spin‐resolved band structures and density of states (DOS) are shown in Figure S3. In the deep valence band region from −8 to −3 eV, where the contribution of Mn orbitals is small, a clear spin splitting is observed with the amount of ∼ 0.5 eV. In contrast, in the low energy bands near the Fermi level from −3 to +3 eV, the Mn *d* orbitals are pronounced, and the spin splitting is not a simple parallel shift, with amounts as large as 4 eV. This large splitting results in several spin‐down band crossings near the Fermi level, leading to strong spin polarization, which is consistent with previous reports [38, 42]. The calculated local magnetic moment is 3.2 *µ*_B_/Mn, in excellent agreement with the experimentally determined saturation magnetization.

We also calculated the magnetocrystalline anisotropy energy (MAE) as Δ*E* = *E_a_*_‐axis_ − *E_c_*_‐axis_ = −115 *µ*eV/Mn, indicating a slight energetic preference for in‐plane magnetization. However, the magnitude of the MAE is small compared to that in typical uniaxial ferromagnets. For example, the kagomé ferromagnet Co_3_Sn_2_S_2_ exhibits an MAE of 183 *µ*eV/Co [43], while heavy‐metal alloys such as CoPt and FePt exhibit much larger values of 0.97 meV/Co and 2.75 meV/Fe [44], respectively. The computed MAE in MnRhP is much smaller, although it is larger than the cubic anisotropy energies of elemental Fe and Ni (∼ 5 *µ*eV/atom) [45]. Experimentally, a small positive uniaxial anisotropy energy constant (*K*_u_ ∼ 168 kJ/m^3^ at 2 K) is observed, as shown in Figure 1f. The discrepancy in sign and the small magnitude of the MAE indicate that the magnetocrystalline anisotropy in MnRhP is intrinsically weak.

**FIGURE 5:**
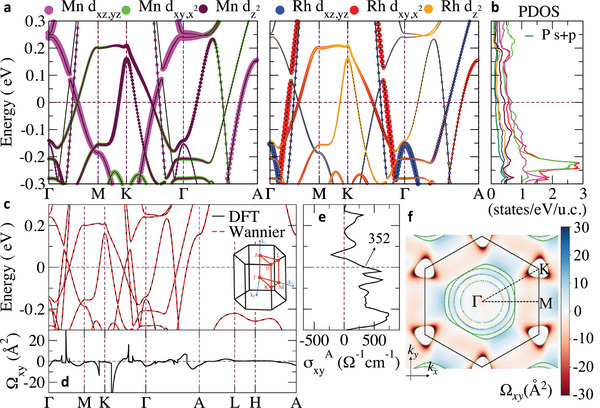
Band structure calculations of MnRhP with SOC. (a) Projected band structures of the *d* orbitals of Mn (left) and Rh (right), *d*_*xz*,*yz*_, $d_{xy,x^{2}-y^{2}}$, and $d_{z^{2}}$, plotted along high‐symmetry paths in the Brillouin zone. (b) Partial density of states (PDOS) for each *d* orbital of Mn and Rh. The total PDOS of P is also shown as a green line. (c) Electronic band structures calculated using DFT and Wannier functions, including Mn *d*, Rh *d*, P *p* orbitals. The inset shows the Brillouin zone. (d) Berry curvature (Ω*_xy_*) distribution along high‐symmetry paths. (e) Anomalous Hall conductivity (AHC) as a function of chemical potential. We obtained *σ_xy_* ^A^ = 352 Ω^−1^cm^−1^ at the Fermi level. (f) 2D mapping of the Berry curvature in the *k_z_* = 0 plane.

The magnitude of the MAE can be explained in terms of the relative SOC strength [46, 47]. For the same spin channel, the SOC matrix elements $\left\langle d_{yz}\left|H_{SOC}\right|d_{xy,x^{2}-y^{2}}\right\rangle$ and $\left\langle d_{yz}\left|H_{SOC}\right|d_{z^{2}}\right\rangle$ prefer in‐plane magnetic anisotropy, while $\langle d_{xy}\left|H_{SOC}\right|d_{xy,x^{2}-y^{2}}\rangle \;$ and 〈*d_yz_* |*H_SOC_* |*d_xz_* 〉 favor out‐of‐plane anisotropy [48]. Therefore, to gain insights into the microscopic origin of the weak anisotropy in MnRhP, we analyzed the orbital‐resolved electronic structure near the Fermi level. The projected band structures of Mn and Rh *d* orbitals are presented in Figure 5a, and the partial DOS (PDOS) for each *d* orbital of Mn and Rh, along with the *s* and *p* orbitals of P, are shown in Figure 5b. Notably, the PDOS at the Fermi level is dominated by contributions from Mn *d*_*xz*,*yz*_, Mn $d_{xy,x^{2}-y^{2}}$ and Rh $d_{xy,x^{2}-y^{2}}$ orbitals, all of which exhibit comparable magnitudes. The coexistence of out‐of‐plane orbital characters (Mn *d*_*xz*,*yz*_) and in‐plane orbital characters (Mn and Rh $d_{xy,x^{2}-y^{2}}$) near the Fermi level leads to a partial cancellation of SOC‐induced anisotropy effects, thus giving rise to the observed weak magnetocrystalline anisotropy.

Figure 5c displays the electronic band structure along high‐symmetry paths in the Brillouin zone (BZ), as illustrated in the inset. The corresponding Berry curvature Ω*_xy_* calculated along the same path is plotted in Figure 5d. Notably, two prominent peaks in Ω*_xy_* are observed near the Γ‐M and Γ‐K directions. Detailed analysis of the band origin of these Berry curvature peaks is given in Figure S4. By shifting the chemical potential, we deduce that gapped nodal lines around the Γ point, as well as the large SOC gap at the K point below and above the Fermi level, are responsible for the Berry curvature peaks.

By comparing Figure 5a,c, we can identify a clear band inversion between Mn and Rh *d* orbitals, which leads to strongly hybridized bands and thus a sizable interband velocity overlap, essentially the numerator of the Berry curvature [49]. Furthermore, the small SOC gap at these band crossings significantly enhances the Berry curvature from the denominator. Moreover, from Figure 5d, due to the lack of band crossings near the Fermi level on the A‐L‐H path, we deduce that the *k_z_* = 0 plane would provide more contribution to the Berry curvature. Thus, the 2D mapping of the Berry curvature in the *k_z_* = 0 plane is given in Figure 5f for a detailed understanding of the relationship between band structures and the Berry curvature distribution. In Figure 5f, we observe that the peak of Berry curvature forms a ring around the Γ point and patches around the K points. Note that MnRhP with an out‐of‐plane magnetic moment retains the horizontal mirror symmetry, which protects nodal lines in the spinless case. With the inclusion of SOC, such nodal lines can be gapped and generate large Berry curvature in a large area of the BZ [50].

Finally, we calculated the intrinsic AHC which is given by the BZ integration of the Berry curvature Ω*_xy_* [49],

(1) $$\begin{array}{c} \;\sigma_{xy}^{A}=-\frac{e^{2}}{\hbar }\sum_{n}\int_{\mathrm{BZ}}\frac{dk}{{\left(2\pi \right)}^{3}}f_{n}\left(k\right)\Omega_{n,xy}\left(k\right) \end{array}$$

where *f_n_* (*k*) is the Fermi‐Dirac distribution function for band *n*. The result is shown in Figure 5e, where the calculated AHC is plotted as a function of chemical potential. At the Fermi level, the AHC reaches a value of *σ_xy_* ^A^ ∼ 352 Ω^−1^cm^−1^, which is in good agreement with the experimental value of *σ_xy_* ^A^ ∼ 280 Ω^−1^cm^−1^ at 2 K. Note that the sign of the AHC is the same as the blue ring around the Γ point in Figure 5f, which is shown as a positive peak of the Berry curvature along the Γ‐M path in Figure 5d. This consistency demonstrates that the large AHC in MnRhP indeed originates from an intrinsic mechanism associated with the Berry curvature induced by topological nodal lines. Moreover, as indicated in Figure S4, interplays between various sets of gapped nodal lines and gapped nodal points in the energy space give rise to the rapid change of the AHC within a small doping region, as we observe in Figure 5e.

Therefore, these computational results provide direct evidence for a link between the symmetry‐protected topology of the band structure and the intrinsic origin of the large AHC in MnRhP.

## 3 Conclusion

We demonstrate that MnRhP is a rare room‐temperature ferromagnet with a distorted kagomé lattice, hosting both topological spin textures and a sizable anomalous Hall response. Our single‐crystal magnetometry and powder neutron diffraction results resolve a previous discrepancy regarding the easy axis in MnRhP, demonstrating that the magnetization aligns strictly along the *c*‐axis rather than being tilted away from it. This clarification provides a reliable basis for understanding and exploiting MnRhP in the context of applications. Furthermore, Lorentz transmission electron microscopy visualizes stable magnetic skyrmions persisting above room temperature, stabilized by the interplay between uniaxial magnetic anisotropy and demagnetization effects. Electrical transport measurements show pronounced temperature‐dependent ordinary and anomalous Hall effects, with the anomalous Hall conductivity reaching 280 Ω^−1^cm^−1^ at 2 K. First‐principles calculations account for the small magnetic anisotropy energy and reproduce the large anomalous Hall conductivity, attributing its origin to the intrinsic mechanism, namely, Berry curvature arising from the gapped nodal lines near the Fermi level. These findings establish MnRhP as an exceptional kagomé ferromagnet, providing a platform to explore room‐temperature topological magnetic and electronic phenomena, as well as potential spintronics functionalities. Moreover, its intrinsically weak magnetic anisotropy suggests that perturbations such as chemical substitution or strain could provide practical routes to tune its properties, offering a promising avenue for the design of functional devices.

## 4 Methods

### 4.1 Sample Preparation

Single crystals of MnRhP were synthesized using a self‐flux method. High‐purity Mn and Rh powders and red phosphorous (P) grains in a molar ratio of Mn: Rh: P = 1.5: 1.5: 1 were loaded into a boron nitride crucible and sealed in an evacuated quartz tube. The mixture was heated slowly to 1180°C over 96 h, then cooled to room temperature. The resulting ingot was crushed, resealed in an evacuated quartz tube, heated to 1150°C for 6 h, and slowly cooled to 1030°C over 60 h. After removing the metal‐rich flux by centrifugation at 1030°C, needle‐like single crystals (∼ 1 mm diameter, ∼ 5 mm length) were obtained (Figure S1a). Residual flux on the crystal surface was removed via mechanical polishing, and crystal orientations were confirmed by X‐ray Laue diffraction (Rigaku, RASCO‐BL II) as shown in Figure S1b. The crystal structure was determined by single crystal X‐ray diffraction using Rigaku XtaLAB Mini II with Mo‐K*α* radiation. The structure was solved with the ShelXT structure solution program using the intrinsic phasing solution method and by using Olex2 as the graphical interface [51]. The model was refined with ShelXL using least squares minimization. Details of the obtained structural parameters are summarized in Tables S1.

For powder neutron diffraction, polycrystalline MnRhP powders with a total mass of 3.2 g were synthesized by solid‐vapor reaction. Stoichiometric amounts of Mn and Rh powders with red phosphorous grains (Mn: Rh: P = 1: 1: 1) were mixed, sealed in an evacuated quartz tube, heated slowly to 900°C for 96 h, then quenched in water. The product was ground into fine powder, resealed, and annealed at 900°C for another 96 h.

### 4.2 Magnetization and Electric Transport Measurements

Magnetization measurements on single crystals were carried out using a superconducting quantum interference device magnetometer (MPMS3, Quantum Design), equipped with a sample rotator for angle‐dependent measurements. The demagnetization factors along the *a*‐axis and *c*‐axis were nearly the same, allowing for a direct comparison of the magnetization curves without applying demagnetization corrections.

Electrical transport measurements, including longitudinal resistivity *ρ_xx_* (*ρ_zz_*) and Hall resistivity *ρ_yx_* (*ρ_zy_*), were conducted on two plate‐like single crystals using a standard five‐terminal method in a physical properties measurement system (PPMS, Quantum Design) equipped with an AC transport option. To eliminate spurious signals due to voltage probe misalignment, the field‐swept data were symmetrized or anti‐symmetrized as follows: *ρ_xx_* (*H*) = [*ρ_xx_* (+*H*) + *ρ_xx_* (−*H*)]/2 and *ρ_yx_* (*H*) = [*ρ_yx_* (+*H*) − *ρ_yx_* (−*H*)]/2.

### 4.3 Powder Neutron Diffraction

Powder neutron diffraction experiments were performed using the DMC diffractometer at the Paul Scherrer Institute (PSI), Switzerland, with a neutron wavelength of 2.454 Å. The polycrystalline MnRhP powders were loaded into a standard cylindrical vanadium container. Diffraction profiles were collected over a broad temperature range (2 K ≤ *T* ≤ 450 K) using a standard cryostat, with no external magnetic field applied during the measurements. Structural refinements and symmetry analysis for both nuclear and magnetic structures were performed using the Mag2Pol software [52].

### 4.4 Lorentz Transmission Electron Microscopy (LTEM)

For the LTEM measurements, thin plates with thicknesses of 100–200 nm were fabricated from a bulk crystal piece using a dual‐beam focused ion beam (FIB) system (Helios 5UX, Thermo Fisher Scientific). Slabs were extracted from the bulk crystal by milling trenches around the region of interest, which were then picked up using in situ micromanipulator (EasyLift) and mounted onto TEM copper meshes. Thinning was then performed subsequently by gradually reducing the ion beam current at 30 kV down to the required thicknesses, followed by final surface cleaning at 2 kV. LTEM measurements were performed above room temperature with a transmission electron microscope (JEM‐2800, JEOL). A specialized single‐tilt heating holder was employed to heat the sample during observation to study the transition of spin textures with temperature change. Out‐of‐plane magnetic fields were applied to the thin plates by adjusting the objective lens current. Quantitative in‐plane induction field maps were obtained by analyzing the under‐and over‐focus LTEM images using the software package QPt (HREM Co., Japan) based on the transport‐of‐intensity‐equation (TIE) [53].

### 4.5 First‐Principles Calculations

The calculations of the electronic structures are conducted using DFT as implemented in the Vienna ab initio simulation package (VASP) [54, 55]. All the calculations are performed using the projector‐augmented wave (PAW) [56] pseudopotential with the generalized gradient approximation (GGA) in the form of Perdew–Burke–Ernzerhof (PBE) [57, 58]. A plane‐wave cutoff value of 500 eV and a Γ‐centered 11 × 11 × 23 *k*‐mesh are used to describe the electronic structure. The valence orbital set is 3*p* ^6^4*s* ^2^3*d* ^5^ for Mn, 4*p* ^6^5*s* ^2^4*d* ^7^ for Rh, and 3*s* ^2^3*p* ^3^ for P, respectively. In the calculations, we use the experimentally determined lattice parameters and atomic coordinates, as listed in Table S1. The band structures are plotted with the help of VASPKIT [59]. Based on the DFT electronic band structures, we construct the Wannier functions using Mn *d*, Rh *d*, and P *p* orbitals. The band structures shown in Figure 5 are calculated with spin‐orbit coupling (SOC), whereas the band structures in Figure S3 are calculated without SOC. For comparison, band structures both with and without SOC are presented in Figure S4. Finally, the AHC is evaluated under the Wannier interpolation [60]. A fine mesh of 150 × 150 × 150 *k* points with an extra adaptive 5 × 5 × 5 *k* mesh is applied during the integration with good convergence.

## Conflicts of Interest

The authors declare no conflicts of interest.

## Supporting information




**Supporting File**: advs73786‐sup‐0001‐SuppMat.docx.

## Data Availability

The data that support the findings of this study are available from the corresponding author upon reasonable request.
