# Supplementary material for: Room‐Temperature Magnetic Skyrmions and Intrinsic Anomalous Hall Effect in a Nodal‐Line Kagomé Ferromagnet MnRhP
Source: Adv Sci (Weinh). 2026 Jan 21;13(17):e21734. doi: 10.1002/advs.202521734 (PMC13042616; doi:10.1002/advs.202521734)
Supplement: Supplementary file 1 — Supporting File: advs73786‐sup‐0001‐SuppMat.docx. [file ADVS-13-e21734-s001.docx]

Supporting Information

**Room-temperature magnetic skyrmions and intrinsic anomalous Hall effect in a nodal-line kagomé ferromagnet MnRhP**

*Kosuke Karube*, Ming-Chun Jiang, Lukas Keller, Jonathan S. White, Yi Ling Chiew, Xiuzhen Yu, Guang-Yu Guo, Ryotaro Arita, Yoshinori Tokura, Yasujiro Taguchi**

K. Karube, M.-C. Jiang, Y. L. Chiew, X. Z. Yu, R. Arita, Y. Tokura, Y. Taguchi

RIKEN Center for Emergent Matter Science (CEMS), Wako, 351-0198, Japan

E-mail: kosuke.karube@riken.jp; y-taguchi@riken.jp

M.-C. Jiang, G.-Y. Guo

Department of Physics and Center for Theoretical Physics, National Taiwan University, Taipei 10617, Taiwan

Physics Division, National Center for Theoretical Sciences, Taipei 10617, Taiwan

L. Keller, J. S. White

Paul Scherrer Institute (PSI), CH-5232, Villigen, Switzerland

R. Arita

Department of Physics, University of Tokyo, Bunkyo-ku, Tokyo, Japan

Y. Tokura

Department of Applied Physics, University of Tokyo, Bunkyo-ku 113-8656, Japan

Tokyo College, University of Tokyo, Bunkyo-ku 113-8656, Japan

**1. Characterization of single crystals**

Single crystals of MnRhP grown by a self-flux method are shown in Figure S1a, exhibiting a needle-like shape along the *c*-axis. Figure S1b presents the X-ray Laue diffraction pattern taken for the (001) cross section, indicating well-defined diffraction spots and confirming the high crystalline quality of the samples. The crystal structure was determined by single-crystal X-ray diffraction, and the obtained structural parameters are summarized in Tables S1.

**
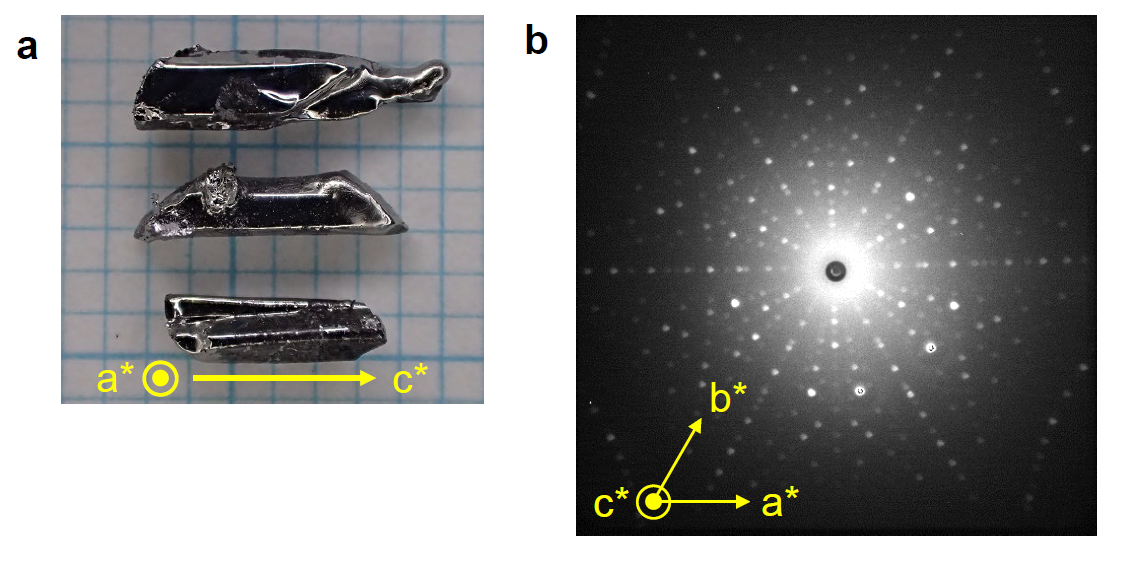
**

**Figure S1.** Characterization of MnRhP single crystals. a) Photo of MnRhP single crystals placed on mm-scale grid paper. b) X-ray Laue back scattering pattern obtained for the *c*-plane.

**Table S1.** Structural parameters of MnRhP determined by single-crystal X-ray diffraction.

| Formula | MnRhP |
| --- | --- |
| Crystal System | hexagonal |
| Space Group | *P*$\bar{6}$2*m* |
| *a* (Å) | 6.2279(7) |
| *c* (Å) | 3.5864(5) |
| *V* (Å^3^) | 120.47(3) |
| *Z* | 3 |
| *ρ*_cal_ (g cm^-3^) | 7.808 |
| *T* (K) | 293(2) |
| *λ* Mo *Kα* (Å) | 0.71073 |
| *μ* (mm^-1^) | 18.478 |
| 2*θ* range for data collection (deg) | 7.556 to 60.262 |
| Measured reflections | 305 |
| Independent reflections | 130 |
| Reflections with *I* > 2*σ*(*I*) | 125 |
| Parameters | 14 |
| *R*_int_ | 0.0091 |
| *R*_1_ / w*R*_2_ [*I* ≥ 2*σ*(*I*)] | 0.0355 / 0.1050 |
| *R*_1_ / w*R*_2_ (all data) | 0.0360 / 0.1055 |

**2. Magnetic states in MnT’X compounds**

We summarize Mn-Mn distances and magnetic ground states in MnT’X (T’= Ru, Rh, Pd; X = P, As) compounds in Table S2 and Fig. S2.^[S1-8]^ The magnetic properties of MnT’X are highly sensitive to the Mn-Mn distance. For example, in the T’ = Rh series, MnRhP with shorter Mn-Mn distances exhibits *c*-axis collinear ferromagnetism, whereas MnRhAs with longer Mn-Mn distances shows antiferromagnetic structures. High-pressure studies further support this trend.^[S8,9]^ However, this trend is opposite in the T’ = Ru and Pd series: Mn(Ru,Pd)P are antiferromagnetic (helimagnetic or spin glass), while Mn(Ru,Pd)As are ferromagnetic. These indicate that, although Mn-Mn distance strongly influences magnetic interactions, the magnetic state is also determined by the combination of T’ and X elements, reflecting their effect on the electronic structure.

**Table S2.** Summary of in-plane (IP) and out-of-plane (OOP) Mn-Mn distances, magnetic ordered states, and magnetic transition temperatures in MnT’X (T’= Ru, Rh, Pd; X = P, As).

| MnT’X | IP Mn-Mn distance (Å) | OOP Mn-Mn distance (Å) | Magnetic ordered state | *T*_C_. *T*_N_  (K) | Ref. |
| --- | --- | --- | --- | --- | --- |
| MnRuP | 3.303 | 3.523 | Helimagnetic* | 269 | [S1-3] |
| MnRhP | 3.281 | 3.586 | Ferromagnetic | 375 | This work, [S4] |
| MnPdP | 3.360 | 3.645 | Spin glass^†^ | 26 | [S4,5] |
| MnRuAs | 3.397 | 3.619 | Ferromagnetic | 496 | [S4] |
| MnRhAs | 3.389 | 3.715 | Antiferromagnetic^‡^ | 240 | [S6-8] |
| MnPdAs | 3.435 | 3.759 | Ferromagnetic | 210 | [S4] |

*An incommensurate helical structure emerges below *T*_N1_ = 269 K. Additional transitions to different incommensurate phases occur at *T*_N2_ = 176 K and *T*_N3_ = 116 K [S1].

†Spin-glass-like freezing behavior is observed below *T*_N_ = 26 K [S4].

‡A canted ferromagnetic structure appears below *T*_C_ = 200 K, followed by a transition to an antiferromagnetic state at *T*_N2_ = 160 K. Another antiferromagnetic phase may exist below *T*_N1_ = 240 K [S6, 8].


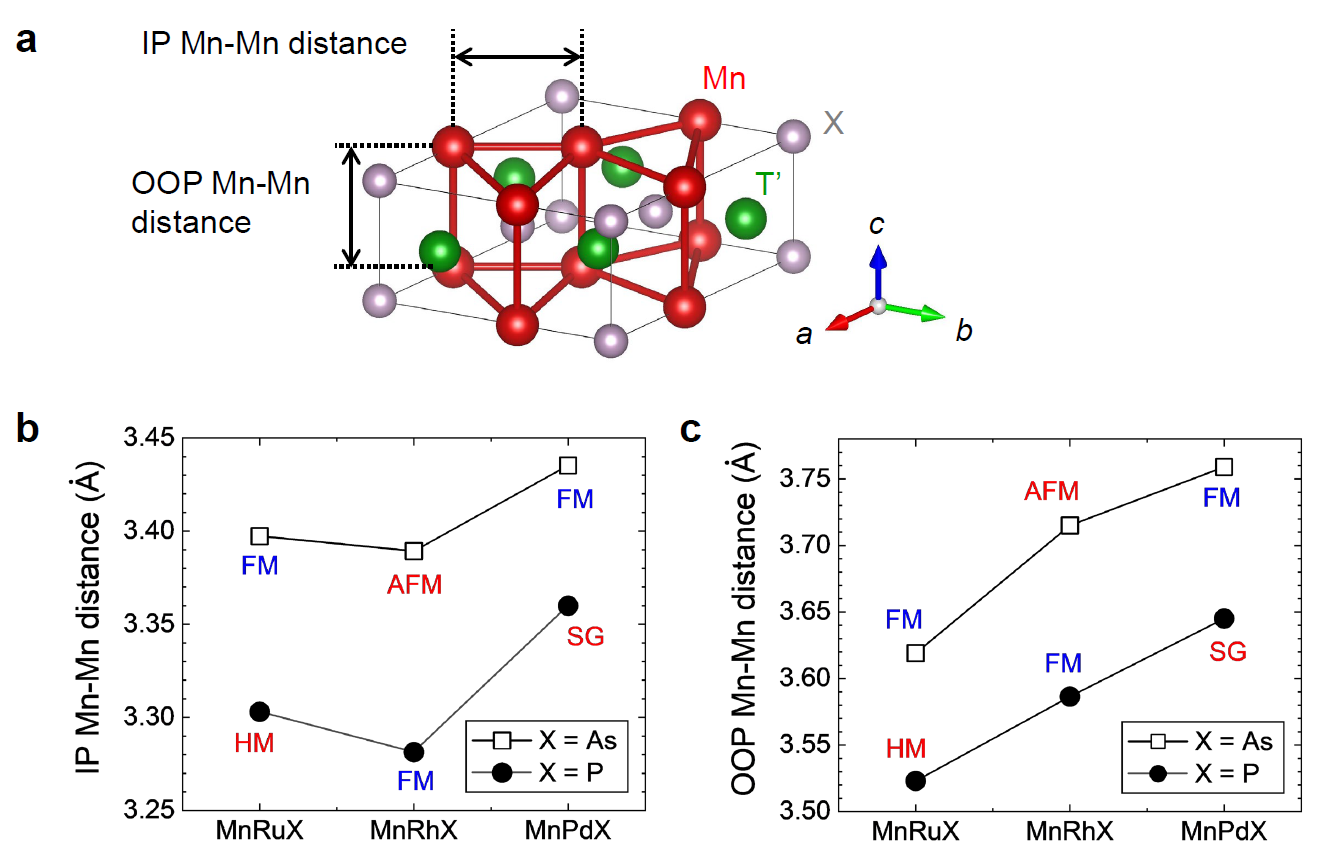


**Figure S2.** a) Schematic of the hexagonal crystal structure of MnT’X. b) In-plane (IP) and c) out-of-plane (OOP) Mn-Mn distances in MnT’X (T’= Ru, Rh, Pd; X = P, As). The magnetic ground states of each compound [ferromagnetic (FM), antiferromagnetic (AFM), helimagnetic (HM), and spin glass (SG)] are also indicated.

**3. Transport properties in the in-plane magnetic field.**

The field dependence of magnetoresistance (MR) Δ*ρ_zz_*/*ρ_zz_*(0) and Hall resistivity *ρ_zy_* in the (*J* || *c*, *H* || *a**) configuration is presented in Figure S3. As shown in Figure S3a, the MR is positive below the saturation field and becomes negative above it. The Hall resistivity *ρ_zy_* is shown in Figure S3b. The anomalous Hall resistivity *ρ_zy_*^A^ was obtained by subtracting the *H*-linear ordinary Hall component. The anomalous Hall conductivity was then calculated as *σ_yz_*^A^ = *ρ_zy_*^A^/(*ρ_yy_ρ_zz_*) and is plotted in Figure S3c.


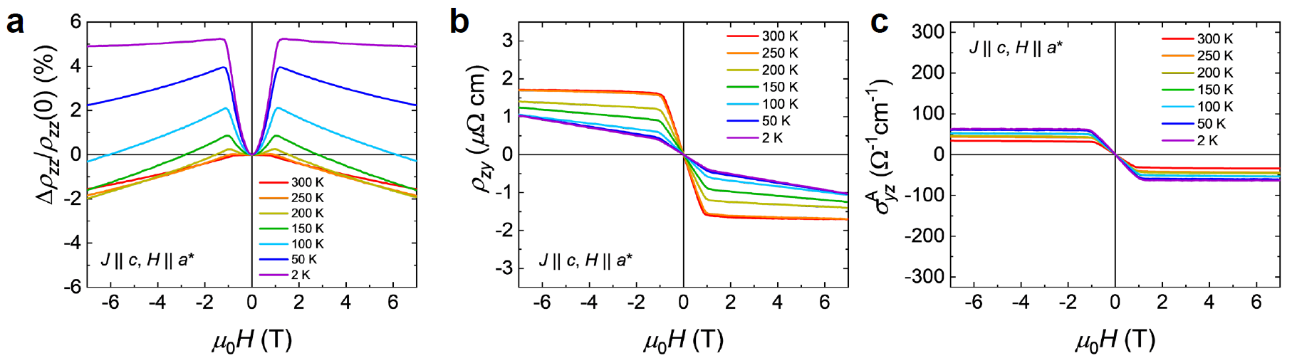


**Figure S3.** Magnetic transport properties under an in-plane magnetic field. a-c) Magnetic field dependence of a) magnetoresistance Δ*ρ_zz_*/*ρ_zz_*(0), b) Hall resistivity *ρ_zy_*, and c) anomalous Hall conductivity *σ_yz_*^A^ measured with current *J* || *c* and magnetic field *H* || *a**.

**4. Spin-resolved band structures.**

The spin-resolved band structures and density of states (DOS) are shown in Figure S4. In the deep valence band region (−8 eV to −3 eV), a spin splitting of ~ 0.5 eV is observed. In contrast, the low-energy region (−3 eV to +3 eV), dominated by Mn *d* orbitals, exhibits a much larger and more complex spin splitting (~ 4 eV). This significant spin splitting results in multiple spin-down band crossings near the Fermi level, leading to strong spin polarization.


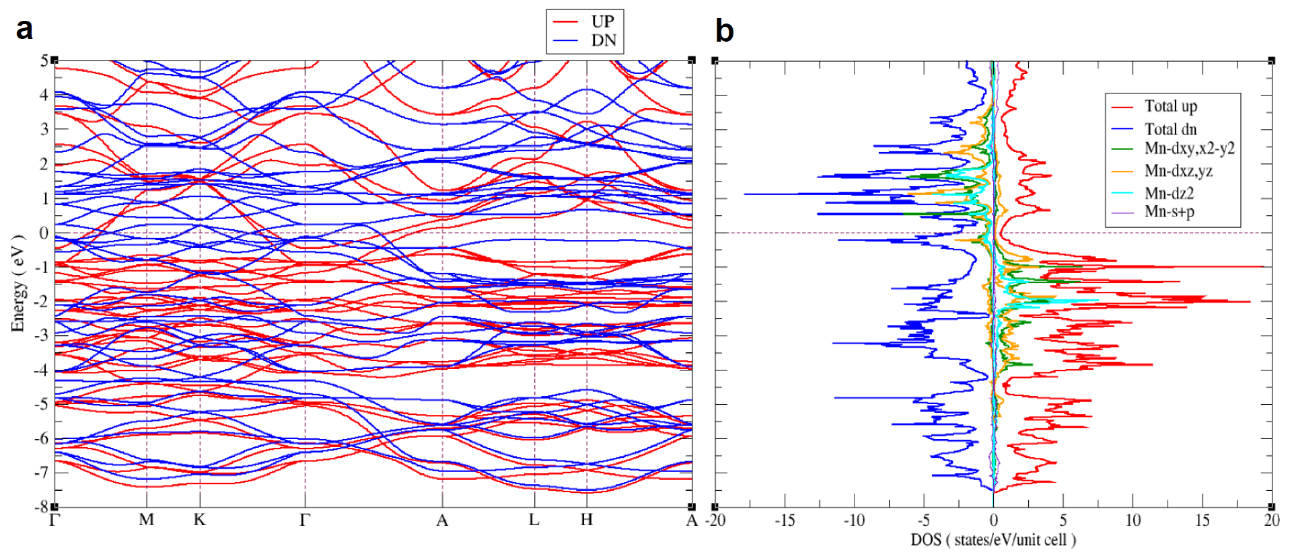


**Figure S4.** Electronic structure of MnRhP in the ferromagnetic state without spin-orbit coupling (SOC). a) Calculated band structures of MnRhP in the ferromagnetic state. Spin-up and spin-down bands are shown in red and blue, respectively. b) Spin-projected density of states (DOS) for spin-up (red) and spin-down (blue) electrons. The Fermi level is set at 0 eV. Flat bands with small dispersion corresponding to Mn *d* electrons appear around −2 eV (spin-up) and 1 eV (spin-down), leading to pronounced peaks in the DOS. Note that the spin direction is opposite to the magnetic dipole moment due to the negative charge of the electrons.

**5. Chemical potential dependance of Berry curvature distribution**

The Berry curvature distribution and its chemical potential dependence along the Γ-M-K line are shown in Figure S5. They are analyzed together with the spin-polarized band structures and the relativistic band structures in Figure S5a and the chemical-potential-dependent AHC in Figure S5b.

First, we focus on the positive Berry curvature peak on the Γ-M line observed in Figure 5d in the main text, which corresponds to the blue circular stripe around the Γ point in Figure 5f. As we shift the Fermi energy down by 0.066 eV (green), an enhancement of the Berry curvature peak is observed in Figure S5c. Judging from the comparison of the spin-polarized and relativistic band structures in Figure S5a with Figure S5c, we conclude that such a peak originates from a SOC-induced gap at a tilted crossing of spin-up and spin-down bands. Since the same crossing is also observed along the K-Γ line, and the Berry curvature peak forms a full circle in Figure 5f, we can deduce that this SOC gap corresponds to a gapped nodal line. Note that Figure S4b indicates that this gapped nodal line gives rise to the largest AHC near the Fermi level with a magnitude of ~ 500 Ω^-1^cm^-1^.

Next, we focus on the Berry curvature peak on the K-Γ line observed in Figure 5d and the red patches around the K point in Figure 5f. As we shift the Fermi energy up from 0 eV (orange) to +0.086 eV (magenta), the negative peak of the Berry curvature in Figure S4c shifts to the left due to the shrinking of Fermi surface, following the downward concave of the band that eventually peaks at the K point. Moreover, the peak intensity of the Berry curvature increases, indicating that the gap openings near the K point are one of the main origins of the Berry curvature in charge neutral. This negative peak reaches its maximum around +0.147 eV (cyan). The anisotropy of the Berry curvature peak (cyan) between the K-M and K-Γ lines arises from the energy difference in the denominator of the Berry curvature expression. Notably, at +0.086 eV (magenta), the largest positive singular peak appears, which also originates from gapped nodal lines that were originally spin-up and spin-down band crossings. However, they are cancelled by the negative peak at the K point, resulting in a nearly vanishing AHC and thus a sign flip of the AHC near this doping region.

In summary, the three black circles in Figure S5a highlight the significant points where the SOC gaps generate Berry curvature peaks near the Fermi level. In particular, the two circles along the Γ-M line demonstrate the presence of gapped nodal lines. Due to the same sign between the large AHC of MnRhP and the Berry curvature peak (green) slightly below the Fermi level, the AHC of MnRhP indeed originates from gapped nodal lines. Gapped nodal lines provide not only large Berry curvature peaks but also large integration areas. Moreover, in MnRhP, the AHC is highly sensitive to doping, reflecting the interplay between various gapped nodal lines and gapped nodal points in the energy space.


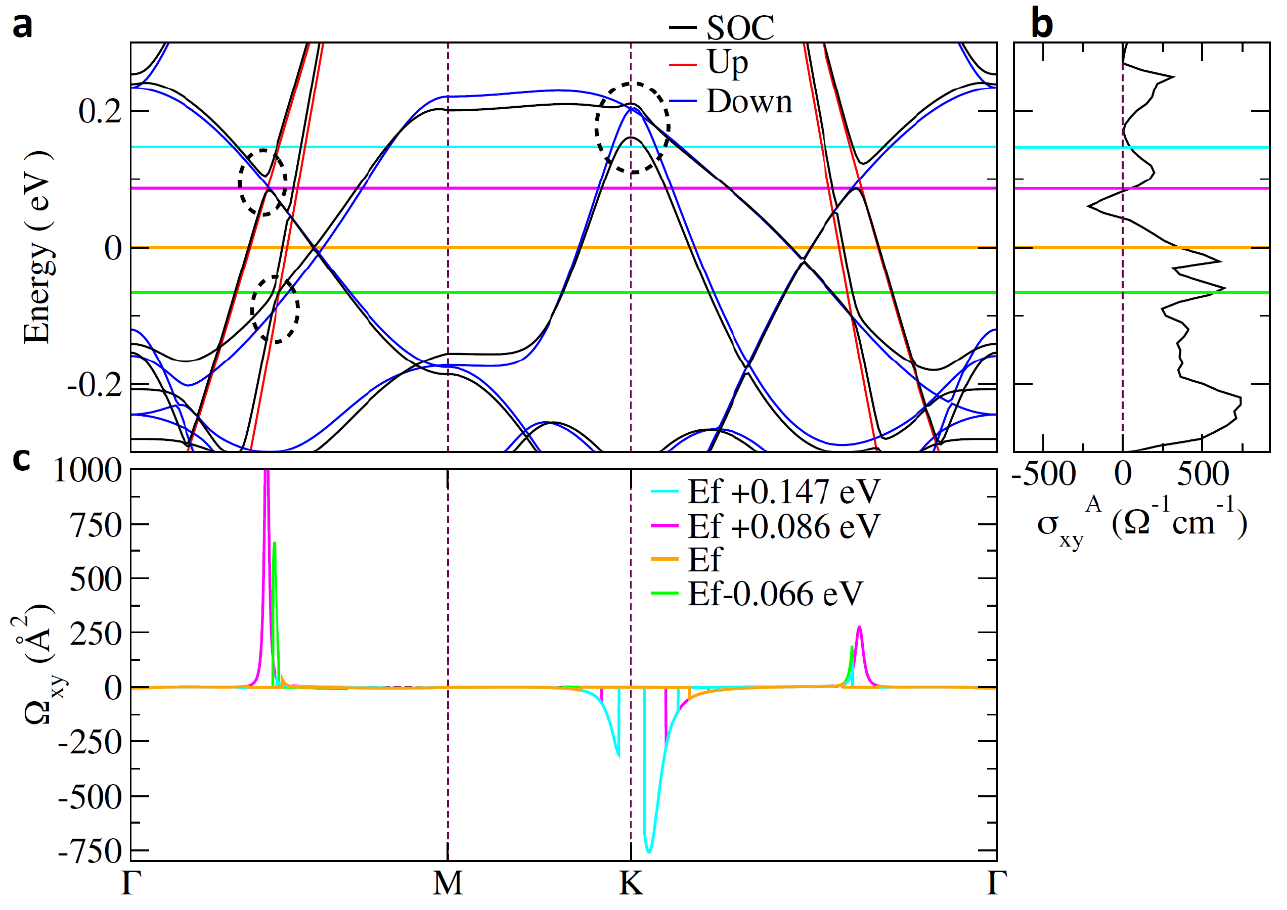


**Figure S5.** Analysis of the chemical-potential dependence of the Berry curvature distribution in ferromagnetic MnRhP. a) Calculated band structures of MnRhP in the ferromagnetic state. Spin-up and spin-down bands without SOC are shown in red and blue, respectively. Relativistic band structures with SOC are shown in black. b) Anomalous Hall conductivity (AHC) as a function of chemical potential. c) Berry curvature (Ω*_xy_*) distribution along high-symmetry paths. The Fermi energy (Ef) is set to 0 eV. Different chemical potentials are shown with Ef +0.147 eV (cyan), Ef +0.086 eV (magenta), Ef (orange), and Ef −0.066 eV (green).

**6. DTF calculation of the anomalous Hall conductivity for in-plane magnetization**

Due to the observation of the sign reversal of the AHC between the out-of-plane and in-plane magnetic field configurations, we also calculated the AHC with the magnetization oriented along the *a*-axis. As shown in Figure S6, the calculated AHC at the Fermi level for the in-plane configuration is *σ_yz_*^A^ = −208 Ω^-1^cm^-1^, which is consistent with the previously reported theoretical result (*σ_yz_*^A^ = −257 Ω^-1^cm^-1^).^[S10]^ These results demonstrate that the DFT, although it overestimates the magnitude, also captures the sign reversal of the AHC between the out-of-plane and in-plane magnetic configurations of MnRhP.


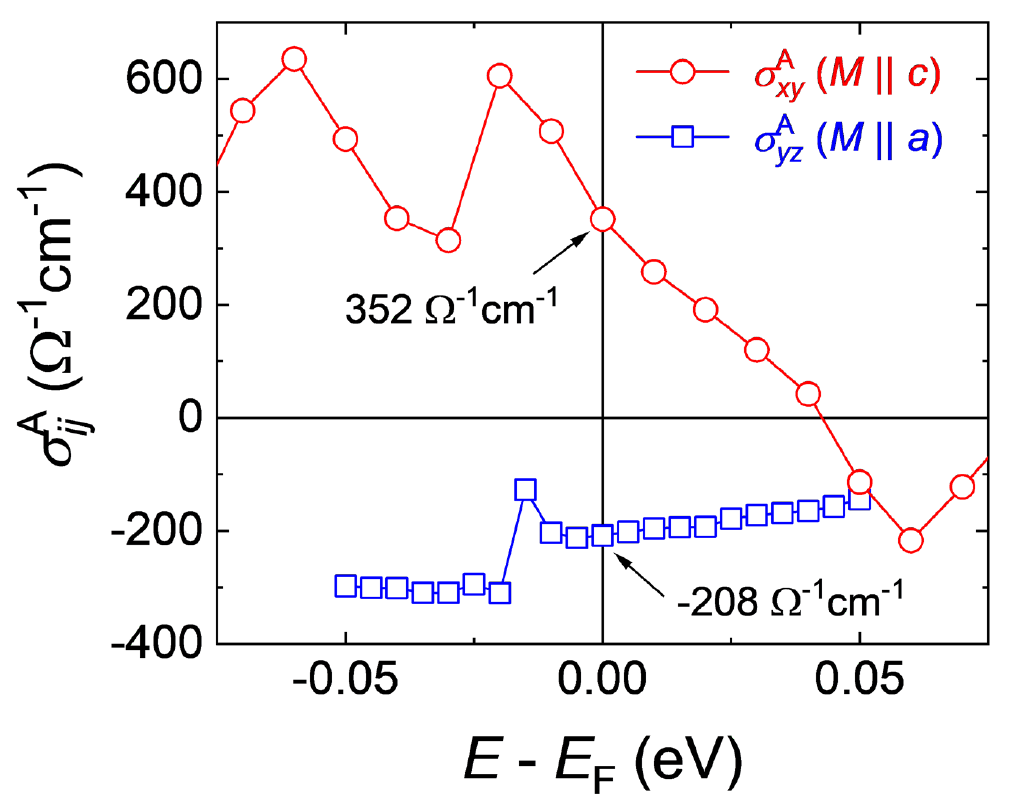


**Figure S6.** Calculated anomalous Hall conductivity as a function of the chemical potential *E* with respect to the Fermi energy (*E*_F_) of MnRhP for magnetization oriented along the *c*-axis (red circles) and *a*-axis (blue squares).

**References**

1. J. Bartolome *et al*., J. Magn. Magn. Mater. **54–57**, 1499 (1986).
2. R. Sereika *et al*., Phys. Rev. B **97**, 214103 (2018).
3. W. Wu *et al*., Phys. Rev. Research **5**, 043133 (2023).
4. T. Kanomata *et al*, J. Appl. Phys. **69**, 4639 (1991).
5. P. Chaudouet *et al*., Proceedings of the International Conference on Solid Compounds of Transition Elements, 7th, 1982, p. IIIA4-1-4.
6. B. Chenevier *et al*., J. Alloys Compd. **179**, 147 (1992).
7. T. Kanomata *et al*. J. Magn. Magn. Mater. **68**, 286 (1987).
8. N. Fujii *et al*., J. Magn. Magn. Mater. **224**, 12 (2001).
9. K. Sato *et al*., J. Magn. Magn. Mater. **177**, 1381 (1998).
10. J. Singh *et al*., J. Phys. Chem. C **126**, 17328 (2022).
